# Estimation of sinking velocity using free-falling dynamically scaled models: Foraminifera as a test case

**DOI:** 10.1242/jeb.230961

**Published:** 2021-02-01

**Authors:** Matthew Walker, Jörg U. Hammel, Fabian Wilde, Tatjana Hoehfurtner, Stuart Humphries, Rudi Schuech

**Affiliations:** 1 School of Life Sciences, Joseph Banks Laboratories, University of Lincoln, Green Lane, Lincoln LN6 7DL, UK; 2 Institute of Materials Research, Helmholtz-Zentrum Geesthacht, Outstation at DESY, Building 66, Notkestr. 85, D-22607 Hamburg, Germany

**Keywords:** Hydrodynamics, Settling, Dynamic scaling, Model, Drag

## Abstract

The velocity of settling particles is an important determinant of distribution in extinct and extant species with passive dispersal mechanisms, such as plants, corals and phytoplankton. Here, we adapted dynamic scaling, borrowed from engineering, to determine settling velocity. Dynamic scaling leverages physical models with relevant dimensionless numbers matched to achieve similar dynamics to the original object. Previous studies have used flumes, wind tunnels or towed models to examine fluid flow around objects with known velocities. Our novel application uses free-falling models to determine the unknown sinking velocity of planktonic Foraminifera – organisms important to our understanding of the Earth's current and historic climate. Using enlarged 3D printed models of microscopic Foraminifera tests, sunk in viscous mineral oil to match their Reynolds numbers and drag coefficients, we predicted sinking velocity of real tests in seawater. This method can be applied to study other settling particles such as plankton, spores or seeds.

## Abstract (precis)

**Summary:** A novel method to determine the sinking velocity of biologically important micro-scale particles using 3D printed scale models.

## INTRODUCTION

The transport of organisms and biologically derived particles through fluid environments strongly influences their spatiotemporal distribution and ecology. In up to a third of terrestrial plants (Willson et al., 1990), reproduction is achieved through passive movement of propagules (e.g. seeds) on the wind. In aquatic environments, propagules of many sessile groups from corals (Jones et al., 2015) to bivalves (Booth, 1983) are dispersed by ambient currents, eventually settling out of the water column to their final locations. Furthermore, most dead aquatic organisms (from diatoms to whales) sink, transporting nutrients to deeper water and contributing to long-term storage of carbon (De La Rocha and Passow, 2007). In the case of microfossils, the sinking dynamics of the original organisms even influences our reconstructions of the Earth's paleoclimate (Van Sebille et al., 2015). Crucially, the horizontal distances over which all these biological entities are transported, and therefore their distribution, are affected by their settling velocity (Ali et al., 2011).

Measuring the individual settling velocity of small particles directly is challenging, especially when they are too small to be imaged easily without magnification (e.g. Walsby and Holland, 2006). Here, we applied dynamic scaling, an approach commonly used in engineering, to circumvent this difficulty and accurately quantify the kinematics of sub-millimetre-scale free-falling particles using enlarged physical models. We used scaled-up physical models in a high-viscosity fluid, enabling easy measurement of settling speed, orientation and other parameters using inexpensive standard high-definition web cameras. While dynamically scaled models have previously been employed to study a number of problems in biological fluid mechanics (e.g. Vogel et al., 1973; Vogel, 1987, 1994; Koehl, 2003), the study of freely falling particles of complex shape – for which settling speed is the key unknown parameter – presents a unique challenge to experimental design that we overcome in this work.

Engineering problems such as aircraft and submarine design often are approached using scaled-down models in wind tunnels or flumes to examine fluid flows around the model and the resulting fluid dynamic forces it is subjected to. To ensure that the behaviour of the model system is an accurate representation of real life, similarity of relevant physical phenomena must be maintained between the two. If certain dimensionless numbers (i.e. ratios of physical quantities such that all dimensional units cancel) that describe the system are equal between the life-size original and the scaled-down model, ‘similitude’ is achieved and all parameters of interest (e.g. velocities and forces) will be proportional between prototype and model (Zohuri, 2015). Intuitively, the model and real object must be geometrically similar (i.e. have the same shape), so that the dimensionless ratio of any length between model and original, Length_model_/Length_real_, is constant – this is the scale factor (*S*) of the model. Less obvious is the additional requirement of dynamic similarity, signifying that the ratios of all relevant forces are constant. For completely immersed objects sinking steadily at terminal velocity (achieved quickly for most small particles, see Materials and Methods, ‘Time to terminal velocity’), dynamic similarity is achieved by matching the Reynolds number (*Re*).

*Re* is a measure of the ratio of inertial to viscous forces in the flow (Batchelor, 2000; within a biological context: Vogel, 1994), and is typically defined as:(1)
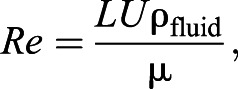
where ρ_fluid_ is the density of the fluid (kg m^−3^); *L* is a characteristic length (m) of the object; *U* is the object's velocity (m s^−1^); and μ is the dynamic viscosity (N s m^−2^, or Pa s) of the fluid. In cases where *LU*ρ_fluid_ is large compared with μ, e.g. whales, birds and fish (*Re*≈3×10^9^ to 3×10^6^; Vogel, 1994), inertial forces dominate. In cases where *LU*ρ_fluid_ is relatively small compared with μ, e.g. sperm, bacteria (*Re*≈3×10^−2^ to 1×10^−5^), viscous forces dominate. Finally, when *LU*ρ_fluid_ is of comparable magnitude to μ, *Re* is intermediate and one cannot discount either inertial or viscous forces. If the scaled model and original system exhibit identical *Re*, the relative importance of inertial versus viscous forces is matched between the two and any qualitative features of the flows (e.g. streamlines) will also be identical.

Dynamically scaled physical models exhibiting the same *Re* as the original systems have been used in a number of biological studies. Vogel and La Barbera (1978) outline the principles of dynamic scaling: to obtain the same *Re* when enlarging small organisms, the fluid flow must be slower and/or the fluid more viscous, and when making smaller models of large organisms, the fluid flow must be faster and/or the fluid less viscous. For instance, Vogel (1987) used air in place of water flowing at lower speeds when investigating the refilling of the squid mantle during swimming by scaling up a model 1.5 times relative to the animal's actual size. More recently, Stadler et al. (2016) investigated sand inhalation in skinks with 3D-printed enlarged models, using helium instead of air (thereby increasing viscosity) as the experimental fluid. Koehl and colleagues have studied crustacean antennule flicking (lobsters: Reidenbach et al., 2008; mantis shrimp: Stacey et al., 2002; and crabs: Waldrop et al., 2015) as well as the movements of copepod appendages (Koehl, 1995) with enlarged models, using mineral oil in place of water. Finally, perhaps the largest change in scale was employed by Kim et al. (2003), who modelled the bundling of *E. coli* flagella at a scale factor of ∼61,000, submerged in silicone oil (10^5^ times more viscous than water), and rotated at 0.002 rpm compared with the 600 rpm observed in real bacteria (Sowa and Berry, 2008).

In all the above studies, basic kinematics such as speed in the original system were relatively easy to measure, and the experiments aimed to reveal the forces involved (e.g. hydrodynamic drag) or details of the fluid flow such as the pattern of streamlines. As the representative speed (*U*) of the original system was known, designing experiments to achieve similitude was relatively straightforward because the *Re* was also known *a priori* – in these cases, the model size, speed and working fluid properties were simply interrelated through *Re* (Eqn 1). For instance, once a working fluid and the model size were chosen, the required towing speed was obvious. However, in the case of sedimentation of small particles (e.g. spores, seeds, plankton), the sinking speed (*U*) is the key unknown. With an unknown sinking speed, the operating *Re* is also unknown, so it is not straightforward to design experiments that achieve similitude with the original system. Here, we present an iterative methodology leveraging 3D printed dynamically scaled models that allows determination of the sinking speed of small objects of arbitrarily complex shape.

We used planktonic organisms of the subphylum Foraminifera (hereafter referred to as the group ‘foraminifera’) as an example of a small (200–1500 µm) biological particle for which the settling velocity is important and typically unknown. Foraminifera are marine amoeboid protists (Gupta, 2002; Schiebel and Hemleben, 2005). By secreting calcium carbonate, foraminifera produce a multi-chambered shell (test) which, in planktonic foraminifera, can grow up to 1500 µm in diameter, and which frequently exhibits a complex shape (Table 1). Once the organism dies or undergoes reproduction, the empty test sinks to the ocean floor, and so oceanic sediment contains substantial numbers of foraminifera tests. Foraminifera account for 23–56% of the oceans’ production of carbonate (CO_3_) (Schiebel, 2002), an important factor in climate change models (Passow and Carlson, 2012). Of particular interest for climate predictions is calculating the flux of tests reaching the ocean floor (Schiebel, 2002; Jonkers and Kučera, 2015). While there are more than 30 extant species and over 600 species in the fossil record, settling velocities are known for only 14 species of foraminifera (Fok-Pun and Komar, 1983; Takahashi and Be, 1984; Caromel et al., 2014: 3.41×10^−4^ to 6.8×10^−2^ m s^−1^, *Re*≈18–55).

**List of symbols and abbreviations**

*A*: particle projected (frontal) area perpendicular to flow
*Ar*: Archimedes number
*C*_D_: coefficient of drag
*C*_D_^E^: interpolating spline through (*Re*,*C*_D_) experimental data
*C*_D_^Ƒ^: *C*_D_ determined through a force balance
*C*_D_^∞^: *C*_D_ in an unbounded domain (i.e. in the ocean)
*C*_D_^walls^: *C*_D_ with walls present (i.e. measured in the tank)
*F*_buoyancy_: buoyant force
*F*_drag_: drag force
*F*_weight_: particle weight
***g***: acceleration due to gravity
*H*: cubic spline interpolant for measured *V* versus *S*
*K*: wall effects correction factor
*L*: maximum length of particle parallel to the flow
*M*: particle mass
*N*: iteration number
^O^: value for real particle at natural operating point
*Re*: Reynolds number
*S*: model scale factor
*U*: sinking speed of particle
*V*: particle volume
*Z*(*t*): depth of the sphere
λ: tank to particle diameter ratio
μ: fluid viscosity
ρ_fluid_: fluid density
ρ_particle_: particle density
Σ*F*: sum of external forces
Ψ: 3D shape

**Table 1.**
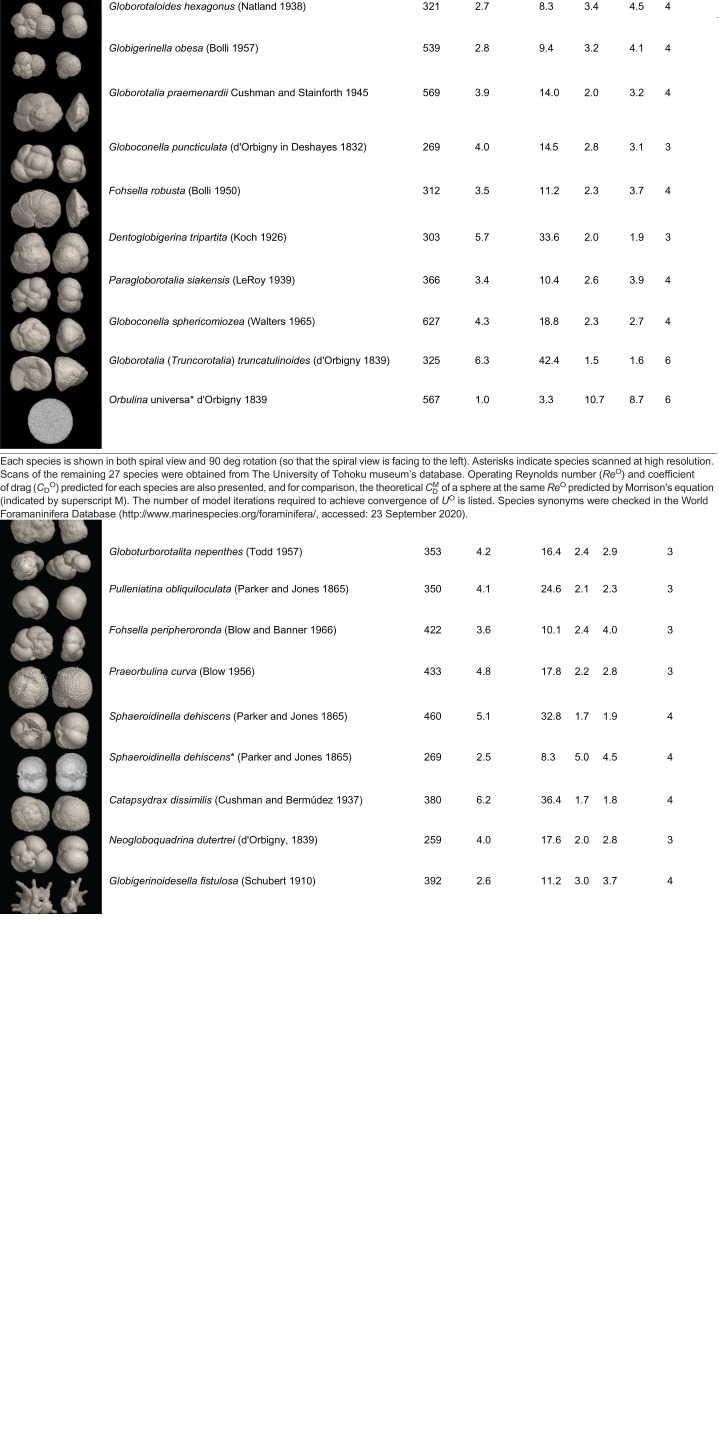
**Predicted sinking speeds *U*^O^ for the 30 species of planktonic foraminifera included in this study**

## MATERIALS AND METHODS

### Similitude and settling theory

We assume that the size (i.e. *L* – defined as the maximum length parallel to the settling direction, *A* – defined as the projected frontal area, and *V* – the particle volume not including any fluid-filled cavities), 3D shape (ψ, here treated as a categorical variable because of our consideration of arbitrarily complex morphologies; see Table 1) and density (ρ_particle_) of the original sinking particle are known, while the sinking speed (*U*) is unknown. The properties of the fluid surrounding the original particle (i.e. ρ_fluid_, μ) are also known, and our goal is to design experiments in which we sink a scaled-up model particle in a working fluid of known ρ_fluid_ and μ in order to determine the model particle's sedimentation speed and, via similitude, *U* of the original particle.

While previous work (Berger and Piper, 1972; Fok-Pun and Komar, 1983; Takahashi and Be, 1984; Caromel et al., 2014) suggests that the *Re* of sinking foraminifera should be 10^0^−10^2^, the exact value of *Re* for morphology ψ is assumed to be unknown. Hence, it is not immediately clear what size the model should be (i.e. the scale factor *S*=*L*_model_/*L*_real_) in order to match this *Re* in the experiments and ensure similitude. Solving for both *Re* and *S* simultaneously requires additional mathematical relationships beyond Eqn 1.

Throughout, we use a superscript O to refer to the original values of dimensioned variables at life size (e.g. *L*^O^, *V*^O^, *A*^O^, 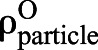, *U*^O^) and *Re*^O^, 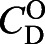 for the values of the dimensionless Reynolds number and drag coefficient (defined below) corresponding to real particles sinking in the original fluid (e.g. seawater of 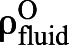, μ^O^). While the fluid dynamics of flow around a particle of particular shape ψ can be considered theoretically over a range of *Re*, only the dynamics at *Re*^O^ and 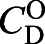 will represent the ‘operating point’ corresponding to the life-size particle settling speed *U*^O^.

**Fig. 1.**
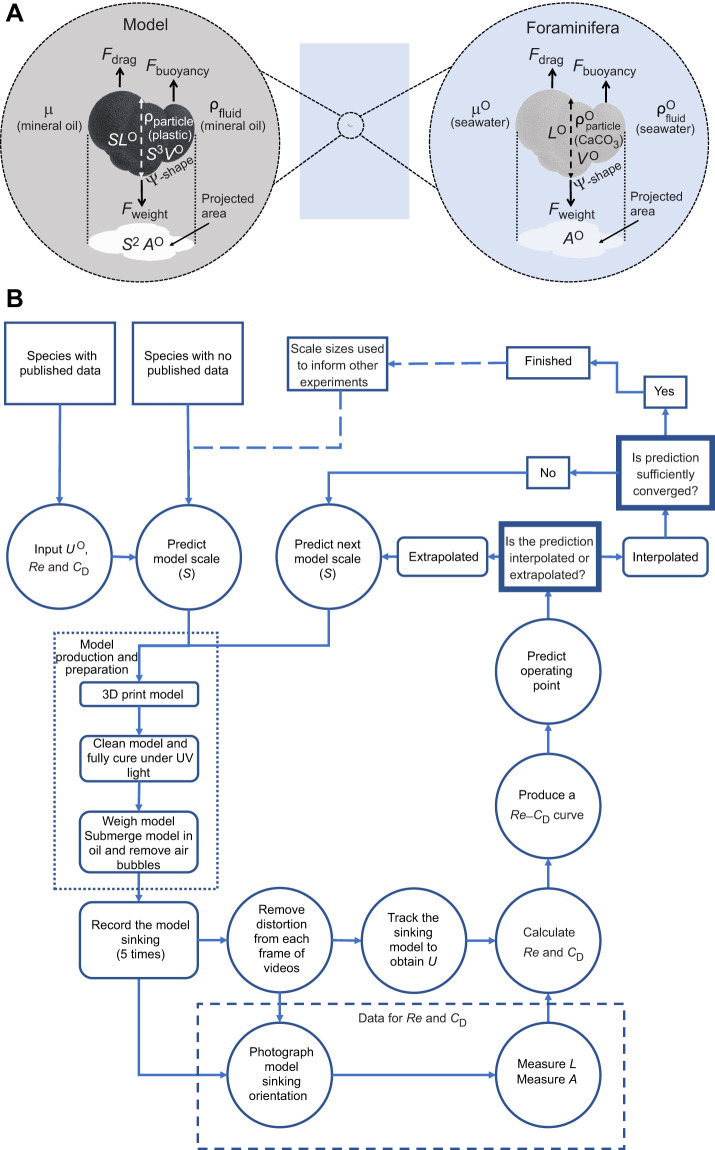
**Model systems and summary of method.** (A) Diagram of relevant forces and parameters between the model (left) and real life (right). (B) Summary of the full method; details are discussed in the main text. Boxes with thicker lines represent a decision, square boxes are data inputs, rounded square boxes are manual processes, and circles are computational steps. *A*^O^, projected area; *C*_D_, drag coefficient; *F*_drag_, drag force; *F*_buoyancy_; buoyancy force; *F*_weight_, particle weight; *L*, maximum length of particle; *Re*, Reynolds number; *S*, model scale factor; *U*, sinking speed of particle; *V*^O^, particle volume; µ, fluid viscosity; ρ_fluid_, density of experimental fluid; ρ_fluid_^O^, density of seawater; ρ_particle_, density of plastic used to make the model; ρ_particle_^O^, density of particle; ψ, particle shape.

When a particle is sinking steadily at its terminal velocity, the sum of the external forces (Σ*F*) acting on the particle is zero (Fig. 1A, Eqn 2); that is, the upward drag force (*F*_drag_, Eqn 3) and buoyant force (*F*_buoyancy_, Eqn 4) must balance the weight of the particle (*F*_weight_, Eqn 5):(2)
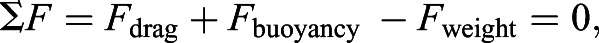
where(3)
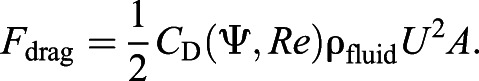
Eqn 3 introduces the drag coefficient *C*_D_(ψ,*Re*), a dimensionless descriptor of how streamlined an object is. Both *C*_D_ and *Re* must be matched to achieve similitude. *C*_D_ depends on the shape of the object ψ, including its orientation relative to the freestream flow; for instance, *C*_D_ of a flat plate oriented parallel to laminar flow is as low as 0.003 while *C*_D_ of a flat plate oriented perpendicular to the flow is ∼2.0 (Munson et al., 1994). However, in addition to object geometry, *C*_D_ also depends on qualitative characteristics of the flow, such as whether it is laminar or turbulent – that is, *C*_D_ also depends on *Re*. *C*_D_ of a sphere decreases from about 200 at *Re*=0.1 to about 0.5 at *Re*=1000; *C*_D_ generally decreases with *Re* for most shapes (Munson et al., 1994; Morrison, 2013). While *C*_D_ does not depend on object size directly, larger objects generally experience higher drag forces and this is captured by the inclusion of particle area (*A*) in the expression for *F*_drag_ (Eqn 3). For brevity, we will omit ψ hereafter and write the drag coefficient as *C*_D_(*Re*).

The buoyant force (*F*_buoyancy_, Eqn 4) and weight (*F*_weight_, Eqn 5) are both expressed using particle volume (*V*), gravitational acceleration (***g***) and density of the fluid (ρ_fluid_) or particle (ρ_particle_), respectively:(4)
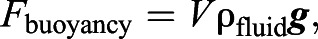
(5)
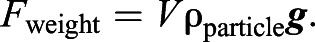
Substituting Eqns 3, 4 and 5 into Eqn 2 and eliminating *U* via the definition of *Re* (Eqn 1) yields an expression for the drag coefficient obtained through a force balance (indicated by a superscript 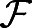):(6)
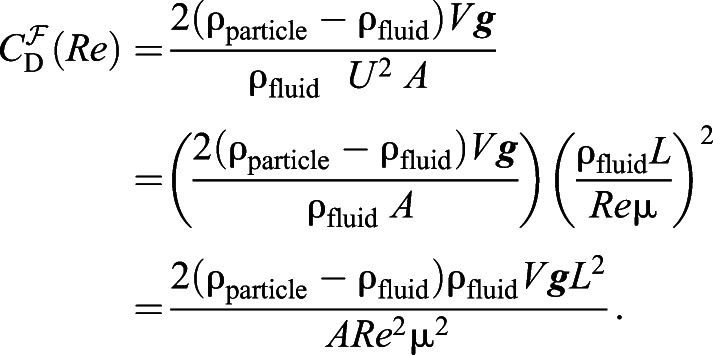
Note that this expression can be simplified further upon identification of the dimensionless Archimedes number *Ar*=***g****L*^3^*_Ar_*ρ_fluid_ (ρ_particle_−ρ_fluid_)/μ^2^ if the cubed length scale *L*^3^*_Ar_=VL^2^*/*A*, yielding 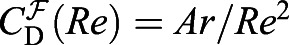, as previously highlighted by others (e.g. Karamanev, 1996). However, we will proceed with the original form of Eqn 6 to keep key variables such as *L* explicit.

If *C*_D_ were known for a particular morphology, we could simply substitute values corresponding to the original test in seawater into Eqn 6 and solve for *Re*=*Re*^O^ and thus *U*^O^ via Eqn 1, immediately solving the problem of unknown settling speed. Unfortunately, the complex shapes of foraminifera (Table 1) coupled with the implicit dependence of *C*_D_ on *Re* means that both variables are generally unknown, and thus far we have only one constraining relationship between *C*_D_ and *Re*. More information is required to determine where along this constraint curve the operating 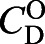 and *Re*^O^ are located. This information can come from experiments in which the sinking speed of scaled-up model particles of various sizes (i.e. scale factors *S*) in a viscous fluid is measured directly, allowing us to calculate *Re* via Eqn 1 and then 
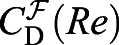
 via Eqn 6 for the models, with appropriate values substituted for each experiment. For clarity, we can rewrite Eqn 6 for a model in terms of *S* and the original test parameters (*L*^O^, *A*^O^, *V*^O^):(7)
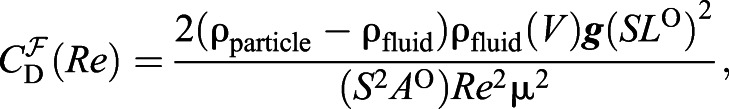
where we use the fact that for a model, *L*=*SL*^O^ and *A*=*S*^2^*A*^O^. While one would also expect *V*=*S*^3^*V*^O^ for 3D printed models, limitations of our 3D printer led to variation in *V* that we overcame using a more general empirical relationship between *S* and *V* based on mass measurements – see ‘3D printer limitations’, below. Eqn 7 represents a constraining relationship between *C*_D_ and *Re* for the sinking particle, which we use to collect (*Re*,*C*_D_) experimental data points at several *S*. Once sufficient data are collected, we can construct a new, empirical relationship (e.g. a cubic spline fit; indicated by a superscript E) between *C*_D_ and *Re* for a particular particle shape, which we term 
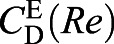
. Finally, we can solve for the operating *Re*^O^, 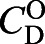, and *U*^O^ by finding the intersection point between the 
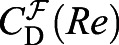
 constraint curve specific to life-size particles sinking in seawater (i.e. Eqn 7 with *S*=1 and 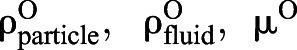) and our empirical 
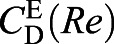
 spline curve valid for a particular particle shape moving steadily through any fluid. MATLAB code can be downloaded from https://github.com/matthewwalkerbio/Dynamic-scaling.

### Study species

To construct an empirical 
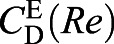
 curve for a particular test morphology, we started with 3D scans of individual specimens from 30 different species (Table 1). The majority of the species were selected from the University of Tohoku museum's database, eForam Stock (http://webdb2.museum.tohoku.ac.jp/e-foram/), with a micro-computed tomography (µCT) scan resolution between 2.5 and 3.6 pixels µm^−1^, and were exported as 3D triangular mesh (STL format) files. Specimens of an additional three species were scanned using synchrotron radiation-based micro-computed tomography (SRµCT). Imaging was performed at the Imaging Beamline P05 (IBL) (Greving et al., 2014; Haibel et al., 2010; Wilde et al., 2016) operated by the Helmholtz-Zentrum-Geesthacht at the storage ring PETRA III (Deutsches Elektronen Synchrotron – DESY, Hamburg, Germany). Specimens were imaged at a photon energy of 14 keV and with a sample to detector distance of 17 mm. For each tomographic scan, 900 projections at equal intervals between 0 and π were recorded. Tomographic reconstruction was done via a classical filtered back projection using the RECLBL library (Huesman et al., 1977). For processing, raw projections were binned 2 times resulting in an effective pixel size of the reconstructed volume of 1.44 µm. These scans were segmented and rendered using SPIERS (Sutton et al., 2012), and again exported in STL format and are available from MorphoSource (https://www.morphosource.org/Detail/ProjectDetail/Show/project_id/1167). Meshes of all foraminifera were manually checked in Meshlab (Callieri et al., 2012) for integrity.

For species where more than one scan was available, the scan that contained the best-preserved specimen was chosen. By only including one specimen per species, this approach neglects phenotypic plasticity which is demonstrated in planktonic foraminifera (e.g. Lohmann, 1983; Morard et al., 2013), but was chosen because of limitations of µCT scan availability and time constraints on the project.

### 3D printing and model preparation

**Fig. 2.**
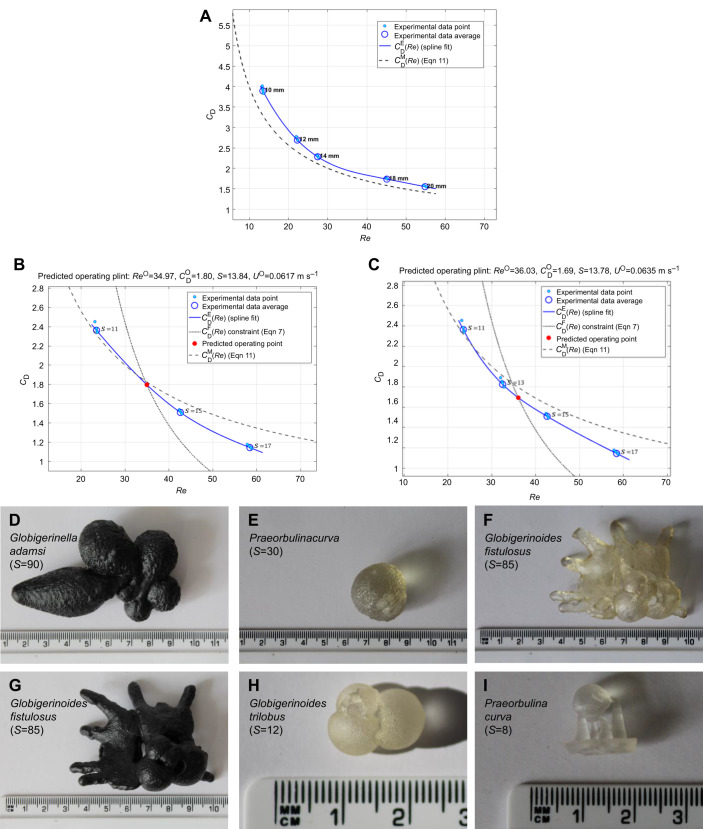
**Application of the method.** (A) Comparison of our empirically generated *C*_D_^E^ curve for 3D printed spheres versus the theoretical *C*_D_^M^ curve (Morrison, 2010). Goodness of fit of *C*_D_^E^ to *C*_D_^M^ is *R*^2^=0.857. Sphere diameter is indicated for each model. (B,C) An example of our iterative solution process for *Catapsydrax dissimilis* showing best estimates of operating values (including the required model scale *S* to achieve similitude) based on experimental data from 3 (B) versus 4 (C) models. For reference, the theoretical *C*_D_^M^ curve (Morrison, 2010) for a sphere is also shown. In B, *S* corresponding to the operating point is estimated as 13.84. After an additional model was sunk at *S*=13 (C), slightly more accurate estimates of the operating *S*, *Re*^O^ and *C*_D_^O^ were obtained. When scaling the model for 3D printing, only 1 decimal place was used rather than the 2 shown. (D–I) Models of foraminifera 3D printed in clear and black resin. Models were used for public engagement but demonstrate the fidelity of the printer compared with the scan data shown Table 1. For definitions, see List of symbols and abbreviations.

The 3D scans allowed us to easily fabricate scaled-up (scale factor *S*) physical models of each specimen using a FormLabs Form1+ (Formlabs, Somerville, MA, USA) 3D printer, using FormLabs Clear Resin Version 2 with a layer thickness of 50 µm (see Fig. 2D–I for examples) and *x*–*y* resolution of 200 µm. Models were washed and flushed with isopropanol to remove excess resin following Formlabs' guide and allowed to air dry. Support material was removed (Fig. 2I), and the models lightly sanded with 400 grit Wet ‘n’ Dry paper, followed by a final isopropanol wash to remove any remaining residue. Once dry, models were filled with mineral oil in preparation for sinking. Clear resin was chosen to allow each model to be checked for bubbles (which would increase the buoyancy of the model). Any bubbles were removed using a 30-gauge needle and syringe.

Following convention, when defining the area *A*_particle_ used in the definition of *C*_D_ (Eqn 3), we measured the projected area of the sinking foraminifera. Referring to high-resolution images of the sinking model (Fig. S2D), a digital model of the foraminifera was manually aligned to measure the projected area in a plane perpendicular to the sinking direction (Fig. S2D). We used the same procedure to measure the maximum length parallel to the flow (*L*) for the calculation of *Re* (Fig. S2D). These choices facilitated objective comparisons of *C*_D_ across morphologically diverse species, to be detailed in a future study.

### 3D printer limitations

Whilst in principle, the volume of a printed model should simply scale according to *V*=*S*^3^*V*^O^, because of inherent limitations of the 3D printer as well as difficulty in removing excess resin from small models, we found that this expectation was usually not satisfied, and weighing the models showed that *M*/ρ_particle_>*S*^3^*V*^O^ where *M* is particle mass (Fig. S2C). Therefore, we estimated *V* of each model by weighing models on an Entris 224-1S mass balance (±0.001 g) and assuming ρ_particle_ was 1121.43±13.73 kg m^−3^, based on the average mass of five 1 cm^3^ cubes of printed resin. Furthermore, whenever a predicted value for *V* at a given scale factor *S* was needed, i.e. in Eqn 7 (see ‘Remaining iterations’ under ‘Iterative approach’, below), we based this on cubic spline interpolation of our *V*(*S*) data for existing models when sufficient data were available, with extrapolation based on cubic scaling of *V*(*S*) if required (see Fig. S2C):(8)
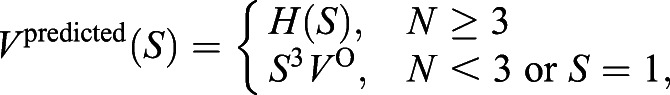
where *N* is the number of existing volume measurements (i.e. the current iteration number) and *H* represents the cubic spline fit of *V* versus *S*. Note that because we always directly measured *V* by weighing after printing each model, and it is not necessary to achieve the exact *Re* and *C*_D_ of the operating point (*Re*^O^ and 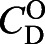) in the experiments (see ‘Remaining iterations’ under ‘Iterative approach’, below), the empirical spline-based volume prediction was not strictly required for our method to succeed. It merely aids in improving the rate of convergence of our iterative approach by reducing the difference between our anticipated and actual *Re*,*C*_D_ for each experiment.

### Settling tank

The models were released in a cylindrical acrylic tank (0.9 m in diameter and 1.2 m in height) of mineral oil (‘Carnation’ white mineral oil, Tennants Distribution Limited, Cheetham, Manchester, UK; ρ=830 kg m^−3^, μ=0.022 Pa S) filled to a depth of 1.18 m (approximately 750 l). The tank was fitted with a custom-designed net and net retrieval system (Fig. S2A) to allow easy retrieval of the models after their descent, allowing each model to be sunk 5 times. Integrated into the net retrieval system was the release mechanism, which was held centrally over the tank, with the grasping parts submerged below the oil level. This ensured that each model was released in a controlled and repeatable fashion.

### Particle imaging

To minimize reflections, the tank was surrounded by a black fabric tent-like structure. This also served as a dark background to facilitate visualization of the model during descent. The tank was illuminated with a single 800 lumen LED spotlight placed beneath it and, as the Formlab Clear Resin is UV-fluorescent, two 20 W ‘Blacklight’ UV fluorescent tubes were placed above the tank.

The sinking models were recorded using two Logitech C920 HD webcams (Logitech, Lausanne, Switzerland), placed at 90 deg to each other (Fig. S2A) and recording at 960 pixels×720 pixels and ∼30 frames s^−1^, allowing monitoring of the position and orientation of the particle in 3D as it fell. As these consumer-grade webcams use a variable frame-rate system, a custom-written MATLAB script was used to initiate camera recording, recording both frames and frame time stamps. Videos were recorded for 500 frames (∼17 s). Sinking velocity was calculated over a central 0.8 m depth range, ensuring the model was at terminal velocity (see ‘Time to terminal velocity’, below) whilst also avoiding end effects which could slow the model as it reached the bottom of the tank. Based on observations of suspended dust, there was no discernible convection in the tank during any trials that might potentially affect sinking velocity. The curved walls of the tank introduced distortion, which was removed using the MATLAB toolbox ‘Camera Calibrator’ (McAndrew, 2004). Pixel size was 1.06 pixels mm^−1^ with a mean reprojection error of 0.5 pixels; therefore, distance measurements (for calculating sinking velocity) were accurate to within 0.5 mm (0.06% of the traversed depth).

### Velocity calculation

Models were tracked in distortion-corrected frames using a modified version of Trackbac (Guadayol et al., 2017; https://zenodo.org/record/45559#.W6z6c2j0nIU). The per-frame centroid coordinates obtained were then paired with the time stamp values recorded to calculate average settling velocity components in 2D for each camera (below, *U_x_* is horizontal speed from camera one, *U_y_* is horizontal speed from camera two, and *U_z_*_,1_ and *U_z_*_,2_ are the vertical speeds corresponding to the two cameras). A resultant velocity magnitude was then calculated for each camera, and these two values were averaged to yield a single estimate for *U* per experiment:(9)
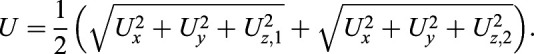
Each model was sunk 5 times and a mean *U* was calculated from these replicates. Replicates beyond a threshold of ±5% of the median sinking velocity were discarded from this average. Each model was dropped one additional time and photographed using a Canon 1200D DSLR camera (Tokyo, Japan) mounted on a tripod close to the tank, to obtain high-resolution (18 megapixels) images which were used to determine model orientation (and thus *L* and *A*) during settling (Fig. S2D).

### Wall effects

At low *Re*, the effects of artificial walls in an experimental (or computational) system can be non-intuitively large and lead to substantial errors if not accounted for (Vogel, 1994). Acting as an additional source of drag, the walls several tens of particle diameters away can slow down a sinking particle and increase its apparent drag coefficient. We designed our experiments to minimize wall effects by using a 0.8 m diameter tank (Fig. S2A) and model diameters of the order of 1 cm. To reduce potential errors further, we applied the method of Fayon and Happel (1960; summarized in Clift et al., 1978) to convert between the apparent drag coefficient when walls are present 
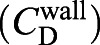
 and the desired drag coefficient in an unbounded domain 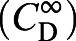:(10)
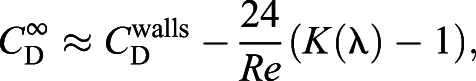
where
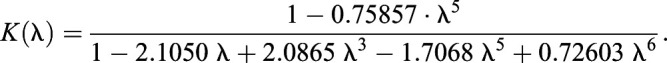
Here, λ=*d*/*D* where *d* is the diameter of the sinking particle and *D* is the tank diameter; we take *d*=*L*. While Eqn 10 is not exact, it substantially reduces the error otherwise incurred if one were to neglect wall effects entirely. Note that Eqn 10 is only valid up to about *Re*=50, beyond which different corrections can be used (Clift et al., 1978).

We applied this correction by taking any experimentally determined *C*_D_ to equal 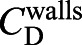, and using 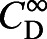 estimated according to Eqn 10 for subsequent calculations as detailed below. In our experiments, λ ranged from 0.0027 to 0.0173, yielding *K* between 1.0057 and 1.0377. Wall effects were therefore quite small, with 
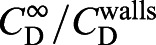
 ranging from 0.993 to 0.994.

### Iterative approach

#### First iteration

To construct an empirical cubic spline 
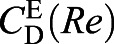
 needed to solve for *U*^O^, at least three experimental data points (corresponding to three scale factors) are needed. These first three *S* were chosen by using an existing empirical *C*_D_(*Re*) relationship for a sphere, valid for 0<*Re*<10^6^ (fig. 8.13, page 625 of Morrison, 2013), indicated by superscript M:(11)
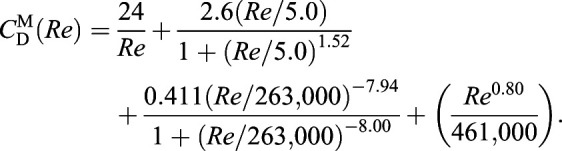


While morphologically complex particles such as foraminifera tests (Table 1) are not expected to behave like ideal spheres, Eqn 11 should be sufficient to provide initial guesses, after which we iterate to find the solution. We note that if the particle shapes of interest were all most similar to some other well-studied geometry (e.g. cylinders, discs, etc.), using a known *C*_D_(*Re*) relationship for that shape could provide better initial guesses and faster convergence.

Substituting Eqn 11 into Eqn 7 (with *S*=1, *V*=*V*^O^, and 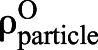, 
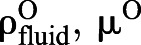
 substituted) and moving all terms to one side, we can numerically solve (MATLAB's *fzero* function) for our first estimate of the operating *Re*^O^. Substituting this *Re* back into Eqn 7 or Eqn 11 yields an estimate of the operating 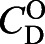. We aimed to reproduce this *Re* and *C*_D_ in the first experiment, except that we accounted for wall effects by distinguishing between 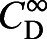 and 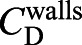 expected to occur in the tank. Hence, we could again substitute this *Re* into Eqn 7 but now with ρ_particle_ corresponding to the resin model and ρ_fluid_ and μ corresponding to mineral oil, and combine this expression with Eqn 10, assuming our estimated *C*_D_=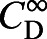, 
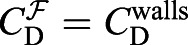
 and λ=*SL*^O^/*D*. The resulting expression can be solved numerically for the first scale factor, termed *S*_1_. Two more scale factors (*S*_2_ and *S*_3_), one smaller and one larger than *S*_1_, were chosen to span expected *Re* values for foraminifera from published literature (e.g. Fok-Pun and Komar, 1983; Takahashi and Be, 1984; Caromel et al., 2014) as well as *Re*^O^ for other species which had reached convergence. This procedure was intended to bound the correct *S* value that reproduces the operating *Re*^O^ and 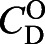 of the settling particle. The three models were printed, their actual volumes *V* measured via weighing, and their settling velocities *U* experimentally measured as detailed in the preceding sections.

An empirical cubic spline curve 
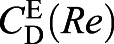
 can now be fitted (http://www.mathworks.co.uk/matlabcentral/fileexchange/24443-slm-shape-language-modeling) to these three initial (*Re*, *C*_D_) data points, constrained to be monotonically decreasing and concave-up within the limits of the data to match expectations for drag on objects at low to moderate *_­_Re*. Three optimally spaced spline knots were used as this yielded excellent fits to the data as the number of data points increased. These details of the spline as well as its order (i.e. cubic versus linear) are somewhat arbitrary but we ensured that our results were sufficiently converged as to be insensitive to them (see ‘Remaining iterations’, below).

The operating point (*Re*^O^, 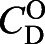) corresponding to the particle settling in the natural environment can be visually represented as the intersection point of the 
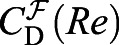
 curve defined by Eqn 7 (with *S*=1 and 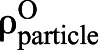, 
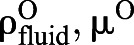
) and the empirical 
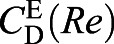
 relationship based on our experimental data. Algebraically, the operating point is the solution to 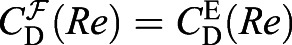. We solved for *Re*^O^ numerically using a root finding algorithm (MATLAB's *fzero* function) on the objective function 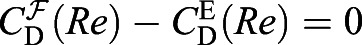 and then obtained 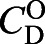 by substituting *Re*^O^ into Eqn 7. Finally, *U*^O^ was easily determined from the definition of *Re*^O^ (Eqn 1 with *U*^O^, L*U*^O^ and 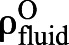 substituted).

Because our first three empirical data points and fitted spline 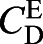 corresponded to guessed model scale factors *S*, our initial operating point prediction (*Re*^O^, 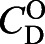) often was not located near any of these initial points or sometimes even within the bounds of these data (in which case linear extrapolation of 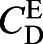 was used to estimate the operating point). Therefore, to ensure the accuracy of our predicted *U*^O^, we continued iterating with additional experiments.

#### Remaining iterations

The model scale factor for the *N*th experiment was chosen by combining Eqns 7, 8 and 10 with *Re*=*Re*^O^ and 
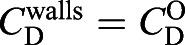
 (from the previous iteration), 
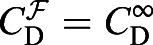
 and *V*=*V*^predicted^, and numerically solving for *S*. A model close to this new scale was printed and sunk, its settling velocity *U* recorded and *Re* and *C*_D_ computed, and a more accurate spline 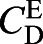 constructed by including this new data point. The calculation of (*Re*^O^, 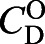) detailed in the previous section was then repeated, yielding a more accurate operating point. Overall, the aim was to tightly bound the predicted operating point with experimental data to maximize confidence in the fitted spline in this region.

The iterative process (visualized as a flowchart, Fig. 1B, with a specific example of convergence given in Fig. 2B,C) was repeated until: (1) the predicted operating point was not extrapolated beyond our existing data, (2) the variation in calculated *U*^O^ between the fitting of a linear spline and cubic spline was no greater than 5%, and (3) the variation between the predicted *Re*^O^ and the closest experimentally measured *Re* was less than 15%.

In many cases, the difference between results based on four versus three data points was very small (Fig. 2B,C), indicating rapid convergence and the possibility of streamlining the method further in the future. Through this method, we calculated the sinking velocity of 30 species of planktonic foraminifera (Table 1).

### Method validation

Our basic methodology was first validated by 3D printing a series of spherical models (10–20 mm in diameter) for which the theoretical *C*_D_(*Re*) relationship is already well known. In order to achieve low density (and thus low sinking velocity and low *Re*), these spheres were hollow and filled with oil via two small holes (of diameter 0.8% of the sphere diameter). Our empirically generated 
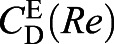
 curve compares favourably with the theoretical 
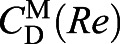
 curve (Morrison, 2010) (*R*^2^=0.875, Fig. 2A), with the distance between the curves approximately constant above *Re*≈25. While the error grows larger at lower *Re*, we expected most foraminifera species to operate at *Re*≈18–55 based on previous work (Berger and Piper, 1972; Fok-Pun and Komar, 1983; Takahashi and Be, 1984; Caromel et al., 2014).

To quantify errors in our approach even more directly, we then considered hypothetical hollow spherical particles with the same material density 
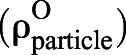
 as foraminifera tests and a range of sizes (*L*^O^=750–1150 µm, similar to the species we studied) settling in seawater. This size range corresponds to *Re*=12–27, the area where our 
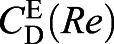
 curve is most divergent from 
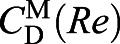
. We compared predictions of the operating *U*^O^ based on our empirical 
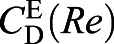
 curve versus the theoretical 
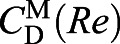
 curve for spheres as outlined above, substituting Eqn 11 for 
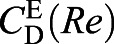
 in the latter case. Maximum relative error in predicted *U*^O^ was 11.5% at *Re*=16 (corresponding to a sphere 860 µm in diameter) while the minimum difference was 6.5% at *Re*=27 (corresponding to a sphere of 1150 µm in diameter, Fig. 2A). This level of error is much smaller than the variation in *U*^O^ we predicted across the 30 foraminifera species we investigated (Table 1).

### Time to terminal velocity

This study was concerned with predicting steady sinking speed, but in our experiments, each model foraminifera took a finite amount of time to accelerate from rest at the point of release to its terminal sinking velocity. As this transient portion of the sinking trajectory could introduce errors into our analysis, it is important to determine whether it affected any of our recorded data.

During the transient acceleration phase, Eqn 2 does not hold. Instead, we can revert to the more general form of Newton's second law:(12)
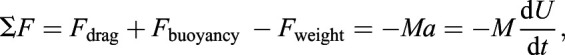
where *M*=*V*ρ_particle_ is particle mass, and the acceleration *a* can be equated to the time derivative of instantaneous velocity d*U*/d*t*. A negative sign appears on the right-hand side of the equation so that we can define the downward movement as positive for convenience. We can then substitute expressions for each force as before:(13)
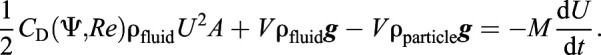
While thus far we have not assumed anything about the particle shape, to proceed further we require knowledge of *C*_D_(Ψ,*Re*) from vanishingly small *Re* (when the particle is at rest) up to the terminal velocity. Hence, we will assume a spherical particle as an approximation to the model foraminifera, so that Morrison's empirical equation (Eqn 11) can then be substituted for *C*_D_(Ψ,*Re*):(14)
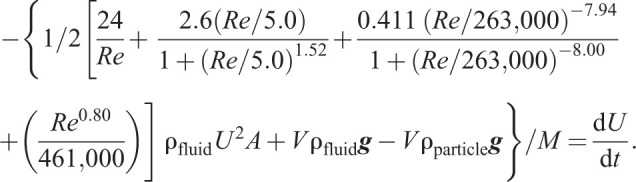


Here, we have isolated d*U*/d*t* on the right-hand side of the equation. If the definition of *Re*=(*LU*ρ_fluid_) /μ is inserted into Eqn 14 (not shown for brevity), one obtains an ordinary differential equation (ODE) for the unsteady velocity *U*(*t*). The depth of the sphere *Z*(*t*) can then be obtained by solving a second much simpler ODE:(15)
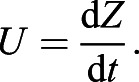
Both ODEs are easily solved numerically by, for example, MATLAB's *ode45* function, subject to the initial conditions *U*(*t*=0)=0 and *Z*(*t*=0)=0.

It is well known that as *Re* approaches zero in the limit of inertia-less Stokes flow, unsteadiness can only occur as a result of time-varying boundary conditions. Thus, a microorganism that stops actively swimming will almost instantly come to a stop, and a heavy micro-particle released from rest will almost instantly begin sinking at its terminal velocity (Purcell, 1977). As *Re* increases and inertia becomes increasingly important, the transient period of acceleration becomes longer. Therefore, a reasonable worst case to examine here is the foraminifera model that sank at the highest *Re*.

We found *Globorotalia* (*Truncorotalia*) *truncatulinoides* to operate at *Re*=42 (Table 1) but here we conservatively chose the largest scale model used to generate its 
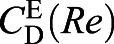
 spline for which *S*=16 and *Re*=90. Inserting this model's length *L*, area *A*, and measured volume *V* into Eqn 14, we obtain solutions for the time-varying speed and depth of a sphere approximating this model's geometry (Fig. S2B). The depth corresponding to where speed equals 99.9% of the terminal velocity is approximately 4.6 cm, which is much smaller than the 19 cm between where the models were released and the edge of the cameras' field of view for data collection. Hence, the transient acceleration of each model foraminifera should have had no effect on our data or results. Most of our models should have reached terminal velocity even sooner as they sank at lower *Re*, e.g. within 2.2 cm for *Catapsydrax dissimilis* operating at *Re*=36 (Table 1).

## RESULTS AND DISCUSSION

Here, we present a novel method of determining settling speed by leveraging dynamically scaled models falling under gravity rather than being towed at a controlled speed. Applying our method to foraminifera-inspired spherical particles (Fig. 2A), we predict settling speeds within 11.5% of theoretical expectations (Fig. S2E). In Fig. 2B,C we present an example of convergence of our method to the operating *Re*^O^, 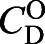 and *U*^O^ of a typical foraminifera species. There was little variation in the number of iterations required to reach convergence (mean 4, range 3–6; see Table 1), despite the morphological complexity of some species (e.g. *Globigerinoidesella fistulosa*). We suspect the higher end of this range was due to these species having forms that were particularly challenging to clean residual resin from, or to the incomplete removal of air bubbles once submerged in oil.

Our predicted sinking speeds of foraminifera fall within aggregated existing data for 14 species (Fig. S1; Fok-Pun and Komar, 1983; Takahashi and Be, 1984; Caromel et al., 2014) and compare well with known speeds for other particles of comparable size and density (e.g. faecal pellets: table 3 of Iversen and Ploug, 2010; phytoplankton: fig. 1 of Smayda, 1971). However, it should be noted that our predicted speeds are higher than published values for five out of the seven foraminifera species for which direct comparisons are possible (Fig. S1). This could be due to our ability to observe enlarged models of sinking foraminifera more accurately compared with actual specimens, and the lack of control for wall effects in previous work, which would tend to underestimate sinking speeds. There could also be considerable natural variation, which our single specimen per species (excluding *Sphaeroidinella dehiscens*) does not capture.

Sedimentation of microscale plankton has been measured both *in situ* (e.g. Waniek et al., 2000) and in the laboratory (e.g. Smayda, 1971; Miklasz and Denny, 2010). By settling dense suspensions of microorganisms, these studies provided a population sinking rate (Bienfang, 1981) which could be 2–3 times lower than the settling velocity of an isolated particle in the typically dilute ocean (Miklasz and Denny, 2010). Other studies have, as in the present study, used enlarged models of microscale plankton to facilitate observations. Padisák et al. (2003) used handmade models of plankton to examine drag, but there was no attempt to accurately match *Re*. Holland (2010) used mechanical pencil leads as models of sinking diatom chains, keeping *Re*<1 in an improvement over Padisák et al. (2003). However, neither study calculated sinking velocity for real organisms. Our dynamic scaling approach ensured that we accurately recreate the fluid flows around settling organisms – a requirement for the correct prediction of sinking speed. We also improved on previous methodologies by effectively eliminating wall effects, basing our models on µCT scans, and using inexpensive cameras to observe natural sinking orientation.

By design, our dynamic scaling approach yields an interpolated *C*_D_(*Re*) curve that describes the flow dynamics (and thus sinking speeds) that would occur if various fluid and/or particle parameters were varied, offering a degree of flexibility not seen in other studies. For example, phytoplankton blooms can increase both the density and viscosity of water due to exudates (Jenkinson et al., 2015), while increasing global temperatures have the opposite effect. The density and viscosity of seawater also naturally vary with latitude. Understanding how these variations affect sinking rates can offer insights into the evolutionary pressures on plankton. Our approach also allows us to isolate the effects of shape on sinking, even across species of widely varying size, density, etc., by comparing *C*_D_ of different species all hypothetically sinking at the same *Re*; a manuscript focused on such biological questions relating to foraminifera is currently in preparation. Differential settling speeds of foraminfera also have implications for nutrient cycling, paleoclimate reconstruction (Kucera, 2007) and the marine calcite budget (Schiebel, 2002).

Our method can easily be modified to study sedimenting particles operating at any *Re*, providing the system's *Re* range can be experimentally replicated. Other sinking marine particles include diatoms (*Re*≈10^−2^ to 1; Botte et al., 2013) and radiolaria (*Re*≈10–200; Takahashi and Honjo, 1983), for which one could use digital models as we have in conjunction with a suitably viscous fluid (high viscosity silicone oil; see Table S1) to enable sufficiently large models to be produced (25 cm; see Table S1). The method can also be applied to terrestrial systems such as settling spores (*Re*≈50; e.g. Gómez-Noguez et al., 2016; Noblin et al., 2009) and dispersing seeds (*Re*≈10^3^; Osuki et al., 2017; Azuma and Yasuda, 1989), again by using 3D printed models based on (often existing) µCT data.

Whilst our method pertains to settling in a quiescent fluid, one could conduct similar experiments using a flume to calculate threshold resuspension velocity (i.e. the horizontal flow speed required to lift a particle off the substrate), important in the study of wind erosion and particle transport and deposition (Bloesch, 1995; Bagnold, 1971). Similarly, studying particles suspended in shear flow could be achieved using a treadmill-like device (e.g. Durham et al., 2009) or a Taylor–Couette apparatus (e.g. Karp-Boss and Jumars, 1998). While additional dimensionless groups beyond *Re* and *C*_D_ would need to be matched to achieve similitude in these systems, we hope that our study provides a starting point for the experimental study of these and other more complex problems.
